# Editorial: External control arms for single-arm studies: methodological considerations and applications

**DOI:** 10.3389/fdsfr.2025.1579171

**Published:** 2025-03-11

**Authors:** Deborah Layton, Laura Hester, Asieh Golozar

**Affiliations:** ^1^ Lane, Clark and Peacock (LCP) LLP, London, United Kingdom; ^2^ Johnson & Johnson, Horsham, PA, United States; ^3^ Nemesis Health, Observational Health Data Sciences and Analytics (OHDSI), New York, NY, United States

**Keywords:** external comparator, methodological innovation, target trial emulation, standardized nomenclature, misclassification bias

## Introduction

An external comparator refers to data from patients outside the parameters of a clinical trial, used to contextualize or compare trial outcomes. These data can come from various real-world data (RWD) sources such as electronic health records, disease registries, or other clinical trials (Mack, 2020; Seeger, 2021; FDA, 2023). External comparators have become important for providing context or comparisons for regulatory submissions in oncology and other rare disease settings when randomised clinical trials (RCTs) are impractical or ethically challenging (Mack, 2020; Seeger, 2021; Mishra-Kalyani, 2022). ECs may also be used in long-term, post-marketing surveillance studies where patients continue on the experimental treatment after the RCT ends (Wang, 2022). The practice of using ECs in support of regulatory and payer submissions as part of evidence for drug approval has also become prevalent in recent years. Patel et al. (2021) identified 433 single-arm clinical trial-based Health Technology Assessment (HTA) submissions between 2011 and 2019, of which 52% contained some type of EC data. As ECs increasingly support regulatory and HTA submissions, methods for these studies need to be advanced to provide the strongest, least biased evidence for approving and reimbursing treatments.

This Research Topic includes five articles that highlight recent methodological developments in EC studies that aim to enhance transparency and improve the robustness of EC studies.

## Standardizing nomenclature and conceptual frameworks

As the use of ECs expands, clear and consistent terminology is essential to ensure methodological transparency and regulatory acceptance. Rippin et al. propose a standardized nomenclature framework for studies comparing clinical trial intervention arms with external data. They emphasize that terminology should reflect the observational nature of EC studies rather than suggest an equivalence to RCT control arms. They caution against the term Externally Controlled Trial unless the EC was pre-specified in the protocol. Instead, they advocate for terms such as External Comparator Cohort (ECC) or External Comparator (EC) when data collection for the EC was not planned before the trial initiation. External patient data used as a comparator differ fundamentally from RCT control arms, as they are drawn from separate populations and often rely on different data collection methods. Also, calling EC populations an “arm” falsely suggests that the external data are directly connected to interventional trial data. The authors advocate that the term “study” is more appropriate since an ECC is a new study being conducted outside the original trial protocol and applies observational study research methods. By adopting precise terminology, researchers and regulators can minimize unrealistic expectations and better align EC studies with observational research principles.

Building upon the need for clearer definitions, Rippin explores the role of estimands in defining treatment effects in EC studies. This perspective article discusses how the estimand framework from the International Council for Harmonisation (ICH) E9 (R1) addendum can be adapted for EC studies. The framework outlines five key estimand attributes: treatment conditions, population, endpoints, handling of intercurrent events, and population-level summary. The author suggests additional considerations for the estimand specific to EC studies, including the baseline definition, the marginal estimator and completeness of data. These attributes are particularly important in EC studies, where data inconsistencies and potential biases require refined methodologies.

## Target trial emulation (TTE): bridging the gap between RCTs and EC studies

A major challenge in EC studies is ensuring methodological rigor to generate regulatory-grade evidence. Arnold et al. propose target trial emulation (TTE) as a framework to improve the design and analysis of EC studies. The TTE framework involves specifying the ideal target trial protocol and then emulating its key elements—such as eligibility criteria, treatment strategies, and outcome definitions—using RWD. This approach enhances comparability between EC and trial cohorts by mimicking RCT design. However, there are some limitations; for example, the effectiveness of the TTE framework relies heavily on the quality and completeness of RWD such that incomplete or inaccurate data can lead to biased results. Unmeasured confounding makes it difficult to draw definitive causal inferences, and implementing the TTE framework can be complex and resource-intensive, requiring detailed knowledge of both the target trial design and the available RWD. Despite these limitations, the transparency and structured principles of TTE can enhance the confidence of the regulatory bodies like the FDA, European Medicines Agency (EMA), and Medicines and Healthcare products Regulatory Agency (MHRA) in EC studies.

## Hybrid study designs: enhancing EC validity in rare diseases

Hybrid natural history studies (NHS), which combine retrospective and prospective data collection, are emerging as a valuable tool in rare disease research, particularly for developing ECAs in clinical trials. Ugoji et al. outline the key design and analysis considerations that are relevant to hybrid designs used as EC, including that these designs must be applicable to or feasible for the target disease and may require additional design and operational considerations to those needed for a standalone retrospective or prospective EC. Given the limited information available in the literature on these designs and the potential for hybrid designs as EC in regulatory submissions, the methodological recommendations in this publication offer a valuable framework for future use of this design, particularly for rare indications.

## Addressing bias in EC studies

A critical challenge in EC studies is bias related to measurement error.

Ackerman et al. explore the impact of measurement error on real-world oncology endpoints, particularly progression-free survival (PFS), focusing on how differences in the assessment methods and timing in real-world data (RWD) limit the comparability of real-world PFS to trial PFS. Two primary sources of measurement bias are identified: misclassification bias and surveillance bias.

Misclassification bias occurs when progression events are incorrectly categorized as false positives or false negatives, distorting PFS estimates. Surveillance bias, stemming from irregular assessment schedules in RWD compared to strict schedules of clinical trials, has a minimal impact on its own but becomes more pronounced when combined with misclassification errors. The findings emphasize that even when trial-derived and real-world PFS estimates appear comparable, underlying biases may persist at the individual level due to incomplete or inconsistent data capture in RWD. To mitigate these biases, the authors recommend improving algorithms for deriving endpoints, conducting simulations to quantify biases, and contextualizing results to account for measurement errors.

The collection of articles in this Research Topic underscores the evolving role of EC studies and the ongoing methodological advancements required to enhance their credibility in regulatory and payer decision-making. By addressing key challenges such as terminology standardization, target trial emulation, hybrid natural history study approaches, and bias mitigation, these contributions provide a strong foundation for future research. As ECs gain wider acceptance among regulatory agencies and HTA bodies, particularly in oncology and rare diseases, ensuring methodological rigor, data quality, and transparency will be crucial for their continued credibility. The sustained collaboration between regulators, industry, and researchers will be essential to refine best practices and establish ECs as a trusted tool for evidence generation, ultimately accelerating access to innovative therapies while maintaining high standards of scientific validity and regulatory confidence.
